# Impact of Arthritis and Multiple Chronic Conditions on Selected Life Domains — United States, 2013

**Published:** 2015-06-05

**Authors:** Jin Qin, Kristina A. Theis, Kamil E. Barbour, Charles G. Helmick, Nancy A. Baker, Teresa J. Brady

**Affiliations:** 1 Division of Population Health, National Center for Chronic Disease Prevention and Health Promotion, CDC; 2 Epidemic Intelligence Service, CDC

About half of U.S. adults have at least one chronic health condition, and the prevalence of multiple (two or more) chronic conditions increased from 21.8% in 2001 to 25.5% in 2012 (1,2). Chronic conditions profoundly affect quality of life, are leading causes of death and disability, and account for 86% of total health care spending (3). Arthritis is a common cause of disability (4), one of the most common chronic conditions (5), and is included in prevalent combinations of multiple chronic conditions (1). To determine the impact of having arthritis alone or as one of multiple chronic conditions on selected important life domains, CDC analyzed data from the 2013 National Health Interview Survey (NHIS). Having one or more chronic conditions was associated with significant and progressively higher prevalences of social participation restriction, serious psychological distress, and work limitations. Adults with arthritis as one of their multiple chronic conditions had higher prevalences of adverse outcomes on all three life domains compared with those with multiple chronic conditions but without arthritis. The high prevalence of arthritis, its common co-occurrence with other chronic conditions, and its significant adverse effect on life domains suggest the importance of considering arthritis in discussions addressing the effect of multiple chronic conditions and interventions needed to reduce that impact among researchers, health care providers, and policy makers.

NHIS uses a complex sampling design to select a representative sample of the U.S. civilian, noninstitutionalized population and collects data continuously throughout the year through the use of in-home interviews. CDC analyzed data from the sample adult component, which includes self-reported data from randomly selected adults (aged ≥18 years) from sampled families. The conditional and final response rates in the sample adult component were 81.7% and 61.2%, respectively.* Doctor-diagnosed arthritis was defined as a “yes” response to the question, “Have you ever been told by a doctor or other health professional that you have some form of arthritis, rheumatoid arthritis, gout, lupus, or fibromyalgia?” Nine other doctor-diagnosed chronic conditions were defined similarly through self-report: hypertension, heart diseases (coronary heart disease, angina pectoris, heart attack, and any other heart condition or heart disease), stroke, diabetes, asthma, cancer, weak or failing kidneys, hepatitis, and chronic obstructive pulmonary disease.† These chronic conditions were selected because 1) they are common comorbidities of arthritis; 2) they are among the leading causes of death and disability; 3) the risks for many conditions can be reduced through modifiable lifestyle factors and public health interventions; and 4) information about them is available in NHIS 2013.

Adults in the 2013 NHIS study population (n = 34,506) were classified into five mutually exclusive categories of chronic condition status: no condition, arthritis only, one nonarthritis condition, ≥2 chronic conditions with one being arthritis, and ≥2 nonarthritis chronic conditions. Six covariates included age group, sex, race/ethnicity, educational attainment level, body mass index (BMI) categories, and smoking status.§ The three life domains selected as outcomes were 1) social participation restriction, defined by answers of “very difficult” or “can’t do at all” to questions¶ about ability to participate in social activities outside the home (specifically, to go shopping, go to movies, go to sporting events; attend clubs, parties, or meetings; or visit friends) and coded as yes/no; 2) serious psychological distress, defined by Kessler 6 scale (6)** and coded as yes/no; and 3) work limitations, defined in two ways as either work disability†† (coded as yes/no) or missing work days during the past 12 months because of illness or injury§§ (coded as 0, 1–5, and 6–365 days).

Prevalence estimates (percentage with 95% confidence intervals [CIs]) were age-standardized using the year 2000 projected U.S. population for the age-groups 18–44, 45–64, and ≥65 years.¶¶ Multivariate binary and multinomial logistic regressions were performed, and predicted marginal proportions (7) were used to estimate prevalences of outcomes adjusting for the six covariates. To examine the role of arthritis, pairwise comparisons of model-adjusted proportions between adults with and without arthritis among those with one or more chronic conditions were performed, using two-sided alpha-level adjusting for multiple comparisons (Bonferroni correction p<0.001). Taylor series linearization method was used to estimate standard errors and account for intracluster correlation because of the complex sampling design.

The overall unadjusted prevalences of chronic conditions were the highest for hypertension (25.6%), arthritis (22.7%), heart diseases (11.4%), diabetes (9.3%), cancer (8.5%), and asthma (7.0%). Among adults with a single nonarthritis chronic condition, the most common conditions were hypertension, asthma, cancer, and heart diseases. Among adults with two or more nonarthritis chronic conditions, the most common multiple chronic condition combinations all included hypertension with diabetes, heart diseases, cancer, or asthma. The most common multiple chronic condition combinations with arthritis included hypertension, heart diseases, diabetes, and cancer (Table 1).

Overall, 51.0% of adults had no selected chronic conditions, 16.8% had one nonarthritis condition, 6.1% had arthritis only, 9.5% had two or more nonarthritis chronic conditions, and 16.6% of adults had arthritis plus other chronic conditions. Among those with arthritis, 73.1% reported additional chronic conditions. Older adults (≥65 years), women, whites or blacks, overweight or obese adults, and current or former smokers were more likely to report having arthritis plus one or more other conditions, compared with younger adults, men, Hispanics, under/healthy weight adults, and never smokers, respectively (Table 2).

The overall unadjusted prevalences of social participation restriction, serious psychological distress, and work disability in the U.S. adult population were 4.2%, 3.8%, and 12.8%, respectively; among those who had jobs in the past 12 months, 33.6% missed 1–5 work days, and 9.9% missed 6–365 work days. Model-adjusted prevalences of all three life domain outcomes increased stepwise among adults with no chronic conditions, one chronic condition (with or without arthritis), two or more nonarthritis conditions, and arthritis plus one or more other chronic conditions, in that order (Table 3). Among adults with one chronic condition, those with arthritis had significantly higher prevalences of social participation restriction and work disability than those with other chronic conditions. Among adults with multiple chronic conditions, those with arthritis had significantly higher prevalences of all the selected life domain outcomes than those without arthritis (Table 3).

## Discussion

Living with multiple chronic health conditions was significantly associated with social participation restriction, serious psychological distress, and work limitations among adults aged ≥18 years, even after adjusting for six important covariates. Arthritis alone had a greater impact on social participation restriction and work disability than having one of the other chronic conditions, and arthritis as one of multiple chronic conditions was associated with higher prevalences of adverse impact on all three life domains. These consequences have profound public health implications because social activity participation, mental health, and the ability to work can be important contributors to quality of life. Missed work days and lost productivity related to chronic diseases are associated with enormous direct and indirect costs, both for those remaining in the workforce and for those who prematurely leave because of disability.***

The findings in this report are subject to at least three limitations. First, because of the cross-sectional nature of NHIS, temporal relationships cannot be established between chronic conditions and outcomes, although causality is plausible for all the associations. Second, NHIS captured only doctor-diagnosed conditions through self-report, which could have led to underreporting of undiagnosed conditions. Finally, the 10 conditions included represent only a subset of chronic conditions.

This study also has several strengths. First, NHIS is a nationally representative data source. Second, because NHIS captures various important chronic conditions, it permits analysis of multiple chronic conditions, an issue of growing importance in the United States. Third, NHIS captures important life domain outcomes. Finally, the large sample size allowed for adequate statistical power when performing multivariate analyses with multiple pairwise comparisons.

What is already known on this topic?

Arthritis is a common cause of disability and often co-occurs with a number of other chronic conditions. Having multiple chronic conditions is a growing problem in the United States, and chronic diseases are leading causes of death, morbidity, disability, and health care costs in the United States.

What is added by this report?

Having an increased number of chronic conditions was linked to adverse outcomes in terms of social participation restriction, serious psychological distress, and work limitations. If arthritis was one of those conditions, the outcomes were even worse.

What are the implications for public health practice?

It is important to include arthritis in discussions addressing the negative effects of multiple chronic conditions and the interventions needed to counter those effects. Inexpensive, proven, but underused strategies can help adults with arthritis and/or other chronic conditions have better quality-of-life outcomes. These strategies include physical activity, maintaining healthy weight, and participating in self-management programs that have been shown to reduce pain and disability, improve function, and address arthritis barriers to physical activity, such as joint pain.

Many chronic conditions not only co-occur, but also share several main risk factors. Modifiable lifestyle behavior factors, such as poor diet, physical inactivity, high BMI, and tobacco use, are some of the root causes of many chronic diseases. Public health interventions and programs that promote healthy diet, physical activity, weight control, and smoking cessation can address these risk factors effectively for individuals and populations (8).

The high prevalence of arthritis, its common co-occurrence with other chronic conditions, and its impact on meaningful life domains suggest the importance of including arthritis in discussions addressing the adverse effects of multiple chronic conditions and the interventions needed to counter those effects among researchers, health care providers, and policy makers. These findings reinforce the need to include public health messages and all-inclusive self-management intervention programs to reduce arthritis-specific barriers to healthy behaviors, such as pain and fear of pain, which can limit physical activity among persons with arthritis.

To address the growing public health burden of multiple chronic conditions, the U.S. Department of Health and Human Services developed a strategic framework to address this problem.††† Effective evidence-based public health interventions, including physical activity, self-management education, and weight loss for overweight/obese adults, help persons with chronic conditions, including arthritis (9,10), manage the negative physical and psychological effects of these diseases, reduce long-term impairment and disability, reduce the need for medical care, improve quality of life, and are cost effective (8). For example, the Chronic Disease Self-Management Program is an intervention that helps persons with a range of chronic conditions. Walk with Ease is an evidence-based program that helps persons with arthritis overcome arthritis-specific barriers to increasing physical activity by learning to walk without increasing joint pain. Improving the availability of these and other evidence-based programs through partnerships with health care systems, worksites, and community organizations can help ensure that tens of millions of persons with chronic conditions can contribute to achieving the *Healthy People 2020* overarching goal to increase quality and years of healthy life.

## Figures and Tables

**TABLE 1:** Percentages of conditions among adults aged ≥18 years with one nonarthritis chronic condition and top six most common chronic condition combinations among those with multiple chronic conditions — National Health Interview Survey, United States, 2013

Rank	1 Nonarthritis chronic condition	%	(95% CI)
1	Hypertension	42.2	(40.6–43.9)
2	Asthma	14.4	(13.3–15.6)
3	Cancer	11.0	(10.0–12.1)
4	Heart diseases	10.9	(9.9–12.0)
5	Diabetes	8.7	(7.8–9.7)
6	Hepatitis	5.4	(4.7–6.1)
7	Chronic obstructive pulmonary disease	5.2	(4.5–6.1)
8	Stroke	1.4	(1.1–1.9)
9	Weak/Failing kidneys	0.8	(0.6–1.2)
**Rank**	**≥2 Nonarthritis chronic conditions** *	**%**	**(95% CI)**

1	Hypertension/Diabetes	15.0	(13.5–16.5)
2	Hypertension/Heart diseases	10.8	(9.4–12.2)
3	Hypertension/Cancer	8.1	(7.0–9.4)
4	Hypertension/Asthma	5.1	(4.2–6.1)
5	Hypertension/Heart diseases/Diabetes	4.2	(3.4–5.2)
6	Hypertension/Heart diseases/Cancer	2.9	(2.3–3.8)
**Rank**	**Arthritis plus ≥1 chronic condition** *	**%**	**(95% CI)**

1	Arthritis/Hypertension	22.0	(20.8–23.3)
2	Arthritis/Hypertension/Heart diseases	7.0	(6.2–7.9)
3	Arthritis/Hypertension/Diabetes	5.7	(5.1–6.4)
4	Arthritis/Cancer	5.2	(4.6–6.0)
5	Arthritis/Heart diseases	5.1	(4.5–5.8)
6	Arthritis/Hypertension/Cancer	4.0	(3.4–4.7)

**Abbreviation:** CI = confidence interval.

* The top six combinations of multiple chronic conditions are presented as a sample of what each category comprised.

**TABLE 2:** Age-standardized distributions among adults aged ≥18 years of five chronic condition categories, by selected characteristics — National Health Interview Survey, United States, 2013

		No chronic condition		1 Nonarthritis chronic condition		Arthritis only		≥2 Nonarthritis chronic conditions		Arthritis plus ≥1 other chronic condition	
						
Characteristic	Sample size*	%	(95% CI)	%	(95% CI)	%	(95% CI)	%	(95% CI)	%	(95% CI)
**Overall** †	**34,505**	**51.0**	**(50.2–51.8)**	**16.8**	**(16.3–17.3)**	**6.1**	**(5.8–6.4)**	**9.5**	**(9.1–9.9)**	**16.6**	**(16.1–17.2)**
**Age group (yrs)** †											
18–44	15,256	74.4	(73.5–75.4)	14.7	(13.9–15.4)	3.7	(3.3–4.1)	3.6	(3.3–4.0)	3.6	(3.2–4.0)
45–64	11,544	38.5	(37.3–39.7)	19.7	(18.8–20.6)	9.2	(8.6–9.9)	12.1	(11.3–12.9)	20.5	(19.6–21.5)
≥65	7,705	14.3	(13.3–15.3)	16.7	(15.7–17.8)	6.4	(5.7–7.2)	19.7	(18.6–20.9)	42.9	(41.4–44.4)
**Sex**											
Men	15,416	55.2	(54.2–56.1)	17.0	(16.2–17.8)	4.5	(4.1–4.9)	9.9	(9.4–10.5)	13.4	(12.8–14.1)
Women	19,089	51.8	(50.9–52.7)	16.1	(15.5–16.8)	7.0	(6.5–7.5)	8.1	(7.6–8.5)	17.0	(16.4–17.7)
**Race/Ethnicity**											
Hispanic	5,938	59.9	(58.5–61.4)	15.9	(14.8–17.1)	4.2	(3.5–4.9)	8.9	(8.0–9.8)	11.1	(10.2–12.1)
White, non-Hispanic	20,769	51.6	(50.8–52.5)	16.7	(16.0–17.4)	6.5	(6.1–6.9)	8.9	(8.4–9.3)	16.3	(15.7–16.8)
Black, non-Hispanic	5,306	49.8	(48.4–51.4)	17.5	(16.3–18.8)	4.9	(4.1–5.7)	10.5	(9.6–11.5)	17.3	(16.2–18.3)
Other	2,492	61.6	(59.4–63.8)	16.5	(14.7–18.4)	4.3	(3.3–5.3)	7.2	(6.0–8.7)	10.4	(8.9–12.1)
**Education level**											
Less than high school	5,351	51.4	(49.7–53.2)	16.4	(15.1–17.9)	4.8	(4.0–5.6)	10.6	(9.6–11.8)	16.8	(15.7–17.9)
High school	8,872	51.8	(50.4–53.1)	16.6	(15.5–17.7)	5.5	(4.9–6.2)	9.5	(8.9–10.3)	16.6	(15.7–17.5)
Some college	10,454	52.0	(50.8–53.2)	15.9	(15.1–16.8)	6.5	(5.9–7.1)	9.0	(8.3–9.7)	16.6	(15.8–17.4)
Completed college or greater	9,669	57.2	(56.1–58.4)	17.1	(16.2–18.1)	6.1	(5.4–6.7)	7.3	(6.8–8.0)	12.3	(11.6–13.1)
**Body mass index category**											
Under/Healthy weight	12,162	62.0	(61.0–63.0)	14.7	(14.0–15.5)	5.9	(5.4–6.5)	6.9	(6.4–7.5)	10.5	(9.8–11.0)
Overweight	11,317	54.9	(54.0–55.9)	16.9	(16.0–17.8)	6.0	(5.5–6.6)	8.4	(7.8–9.0)	13.8	(13.0–14.4)
Obese	9,555	40.5	(39.3–41.7)	18.6	(17.7–19.7)	5.6	(5.0–6.3)	11.7	(11.0–12.5)	23.6	(22.6–24.4)
**Smoking status**											
Current	6,229	47.2	(45.5–48.8)	18.4	(17.0–19.9)	6.2	(5.5–7.1)	10.8	(9.8–12.0)	17.4	(16.1–18.6)
Former	7,642	46.1	(44.5–47.6)	17.9	(16.7–19.2)	7.1	(6.2–8.1)	10.6	(9.8–11.5)	18.3	(17.2–19.4)
Never	20,565	57.6	(56.8–58.4)	15.9	(15.3–16.5)	5.5	(5.1–5.9)	7.6	(7.2–8.1)	13.4	(12.9–14.0)

**Abbreviation:** CI = confidence interval.

* Unweighted number of some variables do not add up to 34,505 because of missing values.

† Not age-standardized.

**TABLE 3:** Model-adjusted* prevalence of adults aged ≥18 years with social participation restriction, serious psychological distress, and work limitations, by chronic condition categories — National Health Interview Survey, United States, 2013

	No chronic condition		1 Nonarthritis chronic condition		Arthritis only		≥2 Nonarthritis chronic conditions		Arthritis plus ≥1 other chronic condition	
					
Life domain	%	(95% CI)	%	(95% CI)	%	(95% CI)	%	(95% CI)	%	(95% CI)
Social participation restriction	1.0	(0.8–1.3)	2.1†	(1.7–2.6)	3.7†	(2.8–4.8)	6.3†	(5.3–7.4)	10.4†	(9.4–11.6)
Serious psychological distress	1.8	(1.6–2.2)	3.5	(2.9–4.2)	3.9	(2.9–5.2)	6.8†	(5.7–8.1)	9.9†	(8.7–11.2)
**Work limitations**										
Work disability	4.3	(3.8–4.8)	8.6†	(7.8–9.5)	15.6†	(13.7–17.6)	22.5†	(20.6–24.4)	30.7†	(29.0–32.4)
**Missing work (days)** §										
None	62.0	(60.9–63.0)	52.9	(50.7–55.1)	48.5	(45.1–52.0)	45.5†	(41.8–49.3)	39.0†	(36.3–41.8)
1–5	30.2	(29.4–31.1)	36.1	(34.6–37.5)	38.6	(36.5–40.6)	40.1†	(38.1–42.1)	42.9†	(41.6–44.2)
6–365	7.8	(7.4–8.3)	11.0	(10.1–12.0)	12.9	(11.4–14.6)	14.4†	(12.6–16.4)	18.1†	(16.3–20.0)

**Abbreviation:** CI = confidence interval.

* Adjusted for age group, sex, race/ethnicity, education attainment level, body mass index category, and smoking status.

† A statistically significant difference of adjusted prevalences is observed between those with and without arthritis among adults with 1 or ≥2 chronic conditions.

§ Among adults who worked or had a job or business with or without pay in the last week or who had a job or business in the past 12 months.

## Footnotes

* Survey description documents are available at http://www.cdc.gov/nchs/nhis/quest_data_related_1997_forward.htm.

† Hypertension, coronary heart disease, angina pectoris, heart attack, and any other heart condition or heart disease, stroke, asthma, cancer, diabetes, hepatitis, chronic obstructive pulmonary disease, and emphysema were ascertained by a “yes” response the question, “Have you ever been told by a doctor or other health professional that you had [condition]?” Weak or failing kidneys and chronic bronchitis were ascertained by a “yes” response to the question, “During the past 12 months, have you been told by a doctor or other health professional that you had [condition]?” Hepatitis was ascertained by a “yes” response to the question, “Have you ever had hepatitis?” For hypertension and asthma, a “yes” response to the following additional questions was used to identify participants with hypertension or asthma: “Were you told on two or more different visits that you had hypertension, also called high blood pressure?” and “Do you still have asthma?” Chronic obstructive pulmonary disease for the analysis was ascertained by a “yes” response to any of the three questions of emphysema, chronic bronchitis, and chronic obstructive pulmonary disease.

§ Smoking status was classified as current, former, or never.

¶ Respondents who answered “very difficult” or “can’t do at all” to either of the following questions were classified as having social participation restriction: “By yourself, and without using any special equipment, how difficult is it for you to…” and ending with “…go out to things like shopping, movies, or sporting events,” and “…participate in social activities such as visiting friends, attending clubs and meetings, or going to parties?”

** Cutoff point of ≥13 in Kessler scale identified respondents with serious psychological distress.

†† Work disability was defined by identifying a person as “unable to work” or “limited in work” when asked if a person was limited in the kind or amount of work.

§§ Missing work days were ascertained by the question, “During the past 12 months, about how many days did you miss work at a job or business because of illness or injury (do not include maternity leave)?” Missing work days were analyzed among adults who worked or had a job or business with or without pay in the last week or who had a job or business in the past 12 months.

¶¶ Additional information available at http://www.cdc.gov/nchs/data/statnt/statnt20.pdf.

*** Additional information available at http://assets1c.milkeninstitute.org/assets/Publication/ResearchReport/PDF/chronic_disease_report.pdf.

††† Additional information available at http://www.hhs.gov/ash/initiatives/mcc/mcc_framework.pdf.
